# Unmodified Gum Arabic/Chitosan/Nanohydroxyapatite Nanocomposite Hydrogels as Potential Scaffolds for Bone Regeneration

**DOI:** 10.3390/polym14153052

**Published:** 2022-07-28

**Authors:** Lara E. Makar, Norhan Nady, Ahmed Abd El-Fattah, Neivin Shawky, Sherif H. Kandil

**Affiliations:** 1 Department of Materials Science, Institute of Graduate Studies and Research, Alexandria University, El-Shatby, Alexandria 21526, Egypt; a_abdelfattah@alexu.edu.eg (A.A.E.-F.); s.kandil@usa.net (S.H.K.); 2 Polymeric Materials Research Department, City of Scientific Research and Technological Applications (SRTA-City), Alexandria 21934, Egypt; 3 Department of Chemistry, College of Science, University of Bahrain, Sakhir P.O. Box 32038, Bahrain; 4 Oral and Maxillofacial Surgery Department, Faculty of Dentistry, Alexandria University, Champollion Street—Azarita, Alexandria 21526, Egypt; noovy60@hotmail.com

**Keywords:** hydrogel, unmodified gum arabic, chitosan, natural nanohydroxyapatite, composite scaffold, acrylic acid, bone regeneration

## Abstract

In this work, physical cross-linking was used to create nanocomposite hydrogels composed of unmodified gum arabic (GA), chitosan (Ch), and natural nanohydroxyapatite (nHA), using an acrylic acid (AA) solvent. Different GA/chitosan contents (15%, 25%, and 35% of the used AA) as well as different nHA contents (2, 5, and 10 wt.%), were used and studied. The natural nHA and the fabricated GA/Ch/nHA nanocomposite hydrogels were characterized using different analysis techniques. Using acrylic acid solvent produced novel hydrogels with compressive strength of 15.43–22.20 MPa which is similar to that of natural cortical bone. The addition of natural nHA to the hydrogels resulted in a significant improvement in the compressive strength of the fabricated hydrogels. In vitro studies of water absorption and degradation—and in vivo studies—confirmed that the nanocomposite hydrogels described here are biodegradable, biocompatible, and facilitate apatite formation while immersed in the simulated body fluid (SBF). In light of these findings, the GA/Ch/nHA nanocomposite hydrogels are recommended for preparing bioactive nanoscaffolds for testing in bone regeneration applications.

## 1. Introduction

Since Hippocrates and Galen, the ability of bone to self-regenerate has prompted study and intrigue [1]. Nevertheless, trauma, inflammation, neoplasm resection, congenital deformity, and degeneration [2,3] remain capable of leaving patients with bone defects beyond a critical size that the body cannot heal. Such patients frequently need invasive surgical intervention to aid healing. As a result, bone is the second most transplanted tissue worldwide, with more than four million surgical procedures involving bone grafts and bone substitute materials performed annually [1]. Even though several solutions have been implemented to address this problem, many clinical demands are still unmet. To date, autologous bone grafts are still the gold standard and most widely considered therapeutic strategy for critical-sized bone defects. This procedure involves transplantation of donor bone from a non-load-bearing site (e.g., the iliac crest) into the defect site in the same patient [4]. However, such autografting has some disadvantages such as excessive costs, limited available tissue from donor sites, and the possibility of postoperative infection of the donor site [3]. Thus, tissue engineering (TE) aims at developing temporary 3D multicomponent scaffolds, to induce the physiological regeneration of functional tissues such as bone, thus avoiding the drawbacks of currently available biomaterials systems [5,6].

The term ”tissue engineering” (TE) is defined by the pioneers Langer and Vacanti as “an interdisciplinary field that applies the principles of engineering and life sciences toward the development of biological substitutes that restore, maintain, or improve tissue or organ function” [5]. Different biomaterials are considered as multicomponent scaffolds; Metallic devices such as plates/screws, rods, and fixators are commonly used, but these are not bioactive or bioresorbable, limiting their performance and necessitating additional surgical procedures in the event of revision. Furthermore, they can cause bone resorption and subsequent implant loosening due to stress shielding effects. Because of their availability and adaptability to a wide range of applications, ceramics are a valuable alternative. Calcium phosphate (CaP) cement, for example, has chemical and functional properties that are very similar to those of bone tissue, and is both biocompatible and bioactive. Low tensile strength and brittleness, on the other hand, are two drawbacks of this material [7]. On the other hand, polymeric scaffolds, according to Khandan et al., 2017, serve as space-filling agents, as delivery vehicles for delivering bioactive peptides, and as three-dimensional structures. They assist in cell organization and provide cues that direct the creation of the appropriate tissue [8]. Polymeric scaffolds, especially hydrogels, are currently a subject of much interest, and their unique configurations and customizable physicochemical properties have been thoroughly investigated. Furthermore, their easy injection into the body eliminates the need for invasive surgical interventions [8]. Nevertheless, the low mechanical strength of synthetic hydrogels limits their applications in certain fields. Tough hydrogels with good mechanical properties that are tensile and have compression strengths in the order of 1–100 MPa and fracture energies in the range of 1–12 kJ m^−2^ are widely studied today, for it remains a crucial task to obtain hydrogels that have high mechanical properties and bioactivity similar to natural bone [9].

Ordinarily, in the formation of such gels, covalent bonds are elaborate between the polymer chains. These covalent bonds usually provide mechanical strength and stability to the gel network [10]. However, covalent bonds are also fragile and irreversible by nature since they undergo permanent alteration and cannot be repaired under normal circumstances. The drawbacks of covalent bonding have hampered the use of hydrogels on an industrial scale. Researchers have addressed this problem by creating supramolecular physical interactions in hydrogels, thereby replacing irreversible connections with reversible bonds [11]. These physical interactions show good self-healing ability in addition to being reversible. Commonly, they are metal–ligand interactions, host–guest interactions, electrostatic interactions, and hydrogen bonds [12]. What has attracted the attention of researchers is the reversible nature of these dynamic supra-molecular connections, which is responsible for the hydrogels’ good self-healing and shear-thinning properties. Amongst these are the non-covalent metal–ligand interaction, due to its thermodynamically stable and kinetically labile nature. In designing self-healing hydrogels, metal ions are used as a powerful species which not only improves the healing behavior of hydrogels but also enhances the reversible property of hydrogels. The mechanical properties of the hydrogels can be modified—depending on the type of metal used—by adjusting the multivalent cations such as divalent cations (Ca^2+^, Sr^2+^, etc.) and trivalent cations (Al^3+^, Fe^3+^, etc.). Because it can also activate some special properties in hydrogels, Fe^3+^ is often utilized to make hydrogels because of its transition nature, which allows it to form complexes more easily than other non-transition trivalent cations. Wei et al., for example, developed physical acrylic acid hydrogels that were ionically cross-linked through ferric ions and exhibited autonomous self-healing behavior due to ferric ion migration [13]. Hence we present a basic yet adaptable method for designing autonomous hydrogel scaffolds in this work.

Chitin is the second most prevalent polymer on the earth, behind cellulose, and is one of the most plentiful organic molecules in nature. Chitin is found in both invertebrates (crustacean shells and insect cuticles) and fungi (mushroom envelopes, green algae, cell walls, and yeasts) [14,15]. Chitosan is a natural polysaccharide consisting of glucosamine and N-acetyl glucosamine with a (1–4) link. It is usually obtained from naturally occurring chitin through chemical deacetylation under heterogeneous conditions (i.e., treatment with alkali at elevated temperature) [16]. Chitosan is biocompatible and can be degraded in vivo without producing toxic byproducts, a fact made evident by the Food and Drug Administration (FDA) approval of chitosan-based drug delivery systems [17]. It is also recognized for its antibacterial characteristics, which make it effective against fungi and bacteria [18]. Nontoxicity, biodegradability, biocompatibility, a heavy metal adsorption effect, an antioxidation effect, film formability, and good bioadhesive properties are only a few of the benefits of chitosan. The disadvantages of chitosan include its low mechanical qualities, processability, and batch variability, all of which limit expansion of its applicability. To address the aforementioned limitations, researchers have combined chitosan with other natural polymers (gelatin, zein, etc.) or synthetic polymers such as poly(vinyl alcohol) (PVA), poly(ethylene oxide) (PEO), etc. Also, chitosan has been blended with inorganic molecules, with or without the further addition of plasticizing agents [19,20]. 

Gum arabic (GA) is a non-toxic, biocompatible, and bio-originated natural polysaccharide polymer that is obtained from the acacia tree. The backbone of GA is formed of 1, 3-linked β-D-galactopyranosyl units having branches of galactose, rhamnose, glucoronic acid, and arabinose residues. It is classified as “Generally Recognized as Safe (GRAS)” by the United States Food and Drug Administration (USFDA). Gum arabic dissolves easily in water, yielding transparent solutions that range in color from very pale yellow to orange-brown and have a pH of ~4.5 [21]. It is frequently utilized as an emulsifier, stabilizer, and thickening agent in food on an industrial scale due to its non-toxic and biocompatible nature. Furthermore, GA is reported to have antioxidant effects [22]. However, it has been discovered that GA cannot produce hydrogel from this basic raw material. Synthetic alteration of GA via interaction with glycidyl methacrylate (GMA) was thought to be a viable solution to this problem. GMA changed GA after the reaction by inserting vinyl groups into the polysaccharide structure of GA. This allowed performing polymerization reaction with hydrophilic vinyl monomers. Currently, vinyl-group-modified GA hydrogels are widely used for hydrogel production [23]. However, converting a GA into a vinyl-modified GA is a lengthy and time-consuming process. Both acrylic acid (AA) and GA can easily be mixed and contain carboxylic groups that produce ionic coordination with Fe^3+^ on deprotonation; further, it can form a considerable number of hydrogen bonds. As a result, GA is employed with the AA monomer without any chemical modifications. The AA and GA are physically cross-linked through FeCl_3_·6H_2_O using the deprotonated functional groups of AA (COO−) and GA (COO− and CO−). GA not only supports the mechanical strength (fracture stress in MPa) of hydrogels but also helps to support the integrity of the gel network. Other positive attributes include high values for stretchability, ductility, toughness, and Young’s modulus at ambient environmental conditions [9].

Strategies to improve the mechanical qualities of hydrogels are widely sought after, so that scaffolds used for bone tissue engineering could be able to provide temporary mechanical integrity at a defect site immediately upon implantation. To decrease or avoid issues such as stress shielding, implant-related osteopenia, and eventual re-fracture, the mechanical properties of scaffolds should be tuned to match the demands of the implant site [24]. It must be also considered that some changes to the hydrogel formulation such as the use of high cross-linking density may lead to toxicity as well as limiting the diffusion of loaded drugs, or the migration of cells and the exchange of gasses and nutrients that are essential for the healing of the fractured bone [25]. Hence, by creating hydrogel composites that combine the biocompatibility and flexibility of the polymeric network with the structural support provided by the filler materials, the mechanical performance of hydrogels can be improved without compromising their positive qualities [26]. Different inorganic/organic composites for BTE have been explored; bioceramics (hydroxyapatite [27], tricalcium phosphate [28]), bioglass particles [29], and carbon nanotubes [30] are the most common fillers employed to date. 

Bioceramics are suited for application in hard tissue replacements due to their chemical and thermal stability, their high strength and abrasion resistance, and their excellent biocompatibility. A compound of great interest in the preparation of bioactive materials is hydroxyapatite, (Ca_10_(PO_4_)_6_(OH)_2_), which is the most thermodynamically stable calcium phosphate salt. The interest in this compound may be due to the fact that it is the inorganic crystalline constituent in calcified hard tissues such as bone and tooth [31]. Natural bovine hydroxyapatite (Ca_10_(PO_4_)_6_(OH)_2_) (HA), as one of the main components of natural bone, can increase the concentration of local Ca^2+^, which can activate the proliferation of osteoblasts and promote the growth and differentiation of mesenchymal stem cells (MSC) [32]. Due to its non-immunogenic properties, biocompatibility, bioactivity, and good bone conductivity, HA has been widely used in bone repair. In addition, it shows a strong affinity to host hard tissues due to its chemical similarity with mineralized human bone tissue [33]. Li et al. developed nHap/polyacrylamide composite hydrogels that exhibited higher fracture tensile stress, higher extensibility, and higher compressive strength (35.8 MPa with 15% nHap vs. 22 MPa for pure gel) in comparison to the parent hydrogels; furthermore, these composites showed excellent shape recovery [34].

Over the past decade, the scientific, engineering, and medical communities have all shown a tremendous interest in three-dimensional (3D) printing. Rapid prototyping and additive manufacturing (AM) are other terms for the 3D printing method, which involves layer-by-layer joining of materials to create items from 3D model data. Customized scaffolds can be created via printing and are extremely desirable for bone tissue engineering. Additionally, at room temperature or at low temperatures, hydrogel precursors made of aqueous solutions of natural polymers such as chitosan, collagen, gelatin, hyaluronic acid, sodium alginate, and polyethelyene glycol diacrylate (PEGDA) could be 3D-printed into scaffolds. These scaffolds could then be further stabilized using UV-, ion-, or temperature-assisted cross-linking. By the use of micro-extrusion, bioceramic particles can be combined with biodegradable synthetic or natural polymers to create 3D scaffolds [35]. The future objective of the researchers is to use this innovative technology with the novel hydrogel that was successfully prepared in this study.

In this work, unmodified gum arabic/chitosan/nanohydroxyapatite (GA/Ch/nHA) novel nanocomposite hydrogels were successfully prepared by free radical addition polymerization and characterized using different analysis techniques such as Fourier transform infrared spectroscopy (FT-IR) analysis, X-ray diffraction (XRD) analysis, energy dispersive X-ray (EDX), and scanning electron microscopy (SEM) imaging. In addition, the GA/Ch/nHA nanocomposite hydrogels were studied for their morphology, mechanical properties, in vitro degradation, apatite forming ability, and in vivo biocompatibility.

## 2. Materials and Methods

### 2.1. Materials

Acrylic acid (AA; purity > 99%) was purchased from Alpha Chemika, Mumbai, India. Gum arabic (GA) (purity > 98%) was obtained from Acros (Geel, Belgium). Chitosan (Mw 100,000–300,000, purity > 99%) was purchased from Bio Basic (Canada Inc.). Ferric chloride hexahydrate (FeCl_3_·6H_2_O, purity > 98%) was purchased from El-Nasr Pharmaceutical Chemicals Company (Oubour, Egypt). Ammonium persulfate (purity > 99%, (NH_4_)_2_S_2_O_8_) (APS) was obtained from Biochem laboratory chemicals (6th of October, Egypt). Sodium phosphate dibasic (purity > 99%, Na_2_HPO_4_), potassium dihydrogen orthophosphate (purity > 98%, KH_2_PO_4_), sodium sulphate (purity > 98%, Na_2_SO_4_), potassium chloride (purity > 99%, KCl), calcium chloride anhydrous (purity > 99%, CaCl_2_), and sodium chloride (purity > 99.5%, NaCl) were obtained from Oxford Lab Chem, Mumbai, India. Sodium bicarbonate was purchased from El Gomhouria Co. Egypt. Magnesium chloride (MgCl_2_·6H_2_O) was purchased from Nice Chemicals Ltd., Kerala, India. 

Cortical femur bovine bone was purchased from a local slaughterhouse which has the approval of the Ministry of Health for clearance of potentially hazardous materials.

### 2.2. Methods 

#### 2.2.1. Preparation of Nano-Hydroxyapatite from Natural Bovine Bone

**Figure 1:**
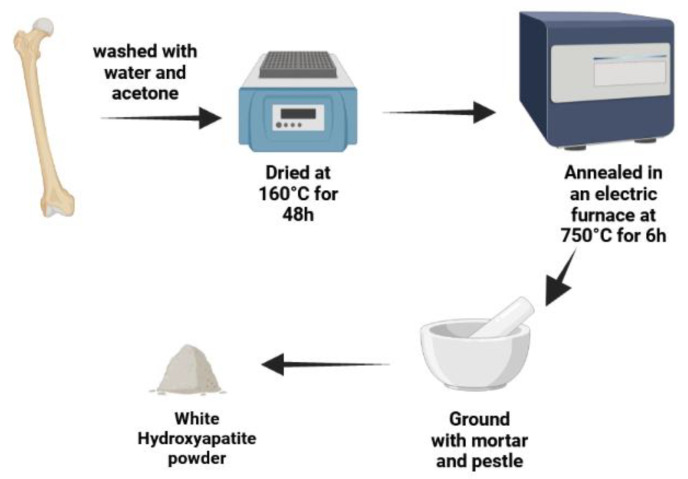
Schematic diagram illustrates the preparation steps of nanohydroxyapatite from natural bovine bone. Created with BioRender.com.

The bovine bone was washed carefully with water and acetone to remove fats and other impurities. Then, the bone was dried at 160 °C for 48 h. The cleaned dried bones were annealed in an electric furnace (Nabertherm 30–3000, Lilienthal, Germany) at 750 °C for 6 h [36]. The bone changed from a brown to white color as shown in Figure 1 (See Supplementary Material Figure S1). Finally, the bone was ground with mortar and pestle to a particle size of less than 450 µm. 

#### 2.2.2. Preparation of Unmodified Gum Arabic/Chitosan/Nano-Hydroxyapatite Nano-Composite Hydrogels

Free radical addition polymerization was used for the physically cross-linked composite hydrogels. In a typical procedure, 30 wt.% AA in distilled water was first prepared in a reaction vessel, followed by the addition of different percentages of GA/chitosan (15%, 25%, and 35% of the used AA). The mixture was stirred until a homogeneous solution was formed. Then, nHA powder (2%, 5%, or 10%) was added to the blend with continuous stirring. After that, 2 wt.% FeCl_3_·6H_2_O was added to the blend as a physical cross-linker with stirring for 2 h. Then, 0.1 g of ammonium persulfate was added to the blend. The blend was placed in the oven for 2 h at 40 °C for complete polymerization. Finally, the hydrogels were kept in a Petri dish for drying at ambient temperature. 

#### 2.2.3. Characterization of the Naturally Prepared Nano-Hydroxyapatite (nHA) and GA/Chitosan/nHA Nanocomposite Hydrogels

##### 2.2.3.1. Energy Dispersive X-ray Spectroscopy (EDX) Analysis

The elements and chemical groups of the prepared nHA were identified using energy dispersive X-Ray spectroscopy (EDX, JEOL IT200-JSM, Tokyo, Japan) The specimens were mounted on aluminum stubs using conductive tape and coated with a gold thin layer for 100 s (Jeol Fine Coat Ion Sputter, JFC-1100E, Peabody, MA, USA). Specimens were evaluated at multiple magnifications with SEM, operating at 20 kV accelerating voltage. 

##### 2.2.3.2. Mechanical Testing

A mechanical measuring system (DMA 7e—Perkin Elmer, Waltham, MA, USA) was used to determine the compressive strength and strain (%) of the GA/chitosan/nHA nanocomposite hydrogels. The cylindrical samples were manufactured with a diameter of 1.5 cm and a thickness of 1 cm. At a strain rate of 0.5 mm/min, a uniaxial compression test was performed. 

##### 2.2.3.3. Scanning Electron Microscopy (SEM) Imaging

Scanning electron microscopy (SEM) (JEOL 5300-JSM, Tokyo, Japan) was used to investigate the microstructure and surface morphology of the nHA and GA/chitosan/nHA nanocomposite hydrogels. Prior to SEM imaging, the samples were sputter-coated with gold and were imaged at a voltage of 20 KV; 100×, 1000×, 3000× and 10,000× magnifications were used. 

##### 2.2.3.4. Fourier Transform Infrared Spectroscopy (FTIR) Analysis

The Fourier transform infrared spectroscopy (FTIR) spectrum for the prepared nHA was recorded throughout the wavenumber range from 4000 to 400 cm^−1^, at a resolution of 4 cm^−1^ using the Perkin-Elmer FTIR spectrometer. 

##### 2.2.3.5. X-ray Diffraction (XRD) Analysis

X-ray diffraction patterns of the prepared nHA, GA/chitosan hydrogel, and GA/chitosan/nHA nanocomposite hydrogel were obtained on a Shimadzu XRD-7000 X-ray (Kyoto, Japan) diffractometer using a CuKα radiation source operating at 40 kV and 30 mA.

##### 2.2.3.6. Water Absorption

Water absorption was determined according to ASTM D570. For each hydrogel, three different discs of 5 cm diameter and 0.5 cm thickness were used. They were immersed in water for predetermined short intervals (5, 10, 15, 30, and 60 min) and one long interval (24 h), the samples were removed from the water, wiped down, and weighed to determine their wet weights (W_w_). Then, they were dried in an oven at 40 °C until they reached a consistent weight and the hydrogel discs were weighed (W_d_). Water absorption is expressed using Equation (1) [37].

(1) $$\mathrm{Water}\;\mathrm{Absorption}\;\left(\%\right)=\frac{\mathrm{Ww}\;-\;\mathrm{Wd}}{\mathrm{Wd}}\;\times \;100$$

##### 2.2.3.7. Degradation Studies

Three samples of each GA/chitosan/nHA hydrogels were immersed in phosphate buffered saline (PBS; pH = 7.4) at 37 °C to study the samples’ in vitro degradation. In sterile glass test tubes, pre-weighted (W_i_) dry samples (hydrogels) were soaked in 5 mL PBS and incubated for 56 days (about 2 months). At 14, 28 and 56 days, the samples were removed from the solution, washed with deionized water, dried at 40 °C until the mass remained unchanged, and measured. After each measurement, each respective tube was replenished with fresh PBS. Finally, the samples’ dry weight (W_d_) was recalculated, and the percentage of biodegradation was measured using Equation (2) [38].

(2) $$\mathrm{Degradation}\;\left(\%\right)=\frac{\mathrm{Wi}-\mathrm{Wd}}{\mathrm{Wd}}\times 100$$

##### 2.2.3.8. Bioactivity Evaluation

The simulated body fluid (SBF, 1 L) with pH 7.4 was prepared according to Table 1. If necessary, the pH was adjusted using hydrochloric acid or sodium hydroxide [39]. The bioactivity of the GA/chitosan/nHA nanocomposite hydrogels was assessed in vitro in simulated body fluid (SBF) at 37 °C, as described in previous study [40]. The hydrogel samples (0.5 mg) were immersed in SBF (5 mL) for 14 days. At the end of the 14 days, the hydrogels were removed from the SBF and dried at 37 °C. The formation of an apatite layer on the surfaces of the hydrogels was evaluated. The microstructure and elemental composition of the hydrogels were examined by the SEM.

##### 2.2.3.9. In Vivo Biocompatibility

**Figure 2:**
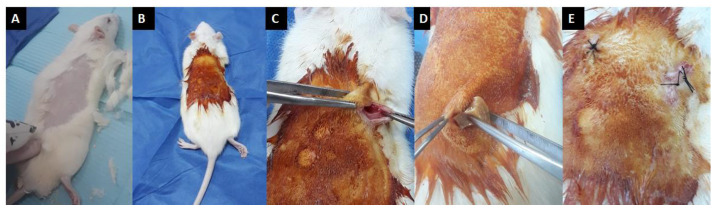
Surgical procedure. (**A**)—shaved rat dorsum, (**B**)—betadine painting of the rat dorsum, (**C**)—a subcutaneous pocket for hydrogel implantation, (**D**)—site of hydrogel implantation, (**E**)—suturing.

Hydrogels were implanted subcutaneously on the dorsal side of Sprague-Dawley rats for 4 weeks. Sterile surgical techniques were applied throughout the experiment. All animal care, pre-, and post-surgical procedures were performed according to the general guidelines of animal welfare of the Institute of Graduate Studies and Research (IGSR), Alexandria University, which comply with the NIH guidelines for the care and use of laboratory animals (NIH Publication #85–23 Rev. 1985) [41]. As shown in Figure 2, the area of implantation was shaved and sterilized with Betadine on the dorsal side of the rat. A transverse incision of approximately 2.5 cm was made in the skin with a sterile number 10 surgical blade and the connective tissue was bluntly dissected to create a subcutaneous pocket. A 25 wt.% scaffold was implanted inside the subcutaneous pocket and the incision was closed with 4.0 sutures. 

The skins of the four rats used were clinically observed for signs and symptoms of inflammation such as redness, hotness and swelling for the duration of 4 weeks. At the end of the 4 weeks, the rat was sacrificed and the area of skin in which the hydrogels were implanted was harvested and fixed in 10 % neutral buffered formalin.

###### Histopathological Evaluation

Hydrogel-implanted skin areas were processed and embedded in paraffin for histological examination. A microtome (Leica RM2156; Leica, Wetzlar, Germany) equipped with a tungsten carbide knife was used to trim and section each block. Hematoxylin and eosin (H and E) were used to stain the sections on the glass slides and morphological evaluation of the tissue response was assessed using a light microscope (Olympus, Japan) [42].

## 3. Results and Discussion

### 3.1. EDX Analysis

**Figure 3:**
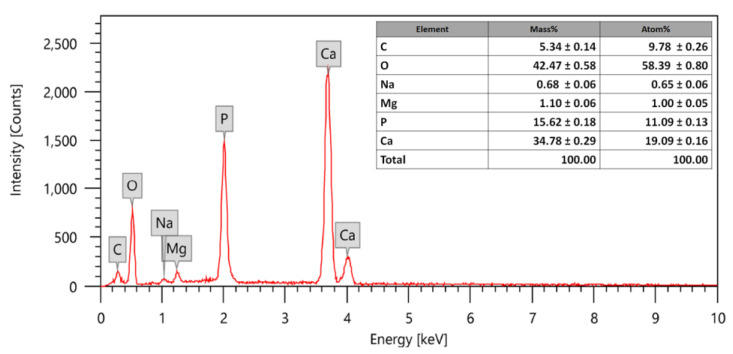
EDX analysis of the prepared nanohydroxyapatite powder.

As shown in Figure 3, the EDX analysis shows evidence of phosphorus, oxygen, and calcium in the hydroxyapatite. In addition, the presented spectrum shows the calcium/phosphorus atomic ratio (Ca/P) of the synthesized nanohydroxyapatite (nHA) to be 1.72, which is very close to the Ca/P ratio of the natural hydroxyapatite in human bone (2.15) [43,44] and to the stoichiometric ratios of Ca and P in synthetic hydroxyapatite (1.667) [45]. The calcium-to-phosphate ratio affects the mechanical properties of the biomaterials and any change in the stoichiometric ratio may lead to an alteration in the mechanical properties of the material [45]. 

### 3.2. Mechanical Properties 

**Figure 4:**
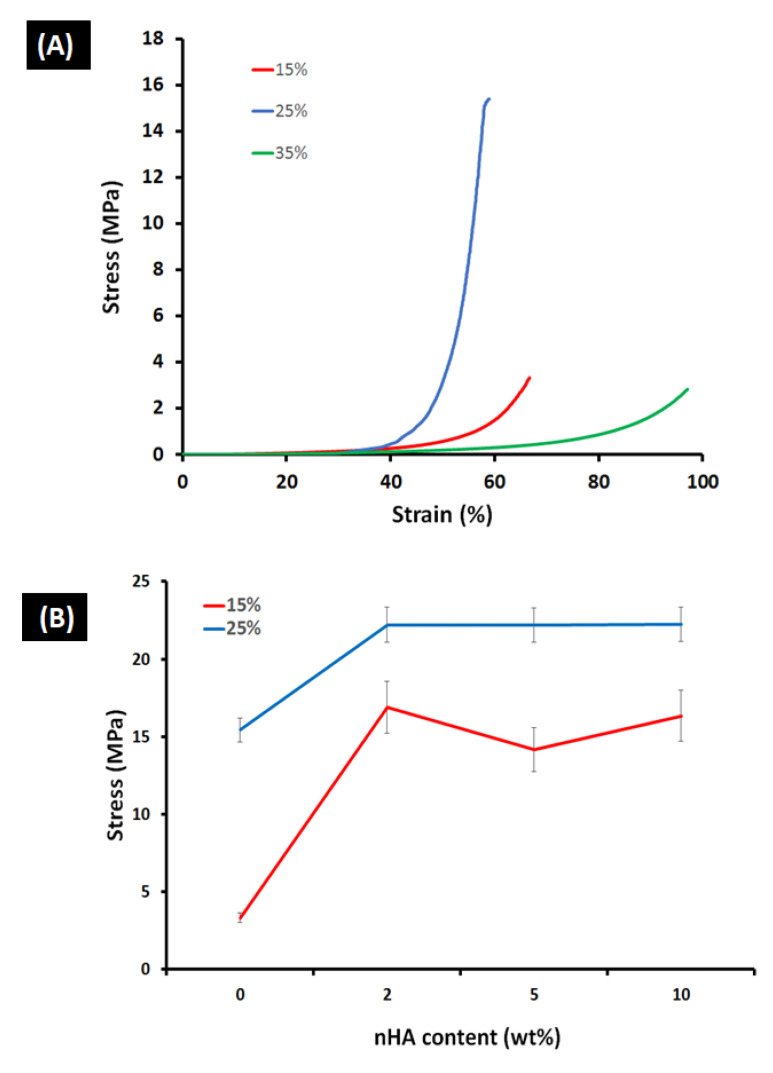
(**A**) Stress–strain compressive strength of the prepared GA/chitosan (15%, 25%, and 35% of the used acrylic acid) hydrogels. (**B**) Stress of 15% and 25% hydrogels as function of nHA content (0, 2, 5, and 10%, respectively).

The compressive strengths of the prepared GA/chitosan (15%, 25%, and 35% of the used AA) hydrogels are shown in Figure 4A. In general, all the prepared hydrogels had compressive strengths from 2.8–15.4 MPa which is similar to compact alveolar bone which has a wide range of 1–100 MPa [46]. The compressive strengths were 3.33 MPa, 15.43 MPa and 2.83 MPa, respectively, for the prepared hydrogels. The 25% GA/chitosan hydrogel sample showed the highest compressive strength of 15.43 MPa, while the 35% hydrogel had the lowest compressive strength. The increase in GA/chitosan from 15 to 25% of AA resulted in an increase in the compressive strength. This can be attributed to an increase in the cross-linking density among the COO− and CO− functional groups with Fe^3+^. However, beyond the 25% concentration, the compression strength decreased. This can be attributed to the aggregation of the molecules of the hydrogel network (i.e., the formation of big clusters in the cross-linked network) that resulted in formation of a non-homogenous network and reduction in the compressive strength [47]. The heterogeneity of the hydrogels may create defects that act as stress concentrators to a much greater extent than with homogenous hydrogels. The 35% concentration was subsequently excluded from this study, due to difficulty in its preparation and poor mechanical performance.

Figure 4B illustrates the effect of nHA content on the compressive strength of the 15% and 25% composite scaffolds. In general, the compressive strength of the pristine hydrogel increased with the increase in the nHA content. As shown in Figure 4B (see Supplementary Figure S2), the compressive strength of the 15% hydrogel increased from 3.33 MPa to, 16.89 MPa, 14.18 MPa, and 16.34 MPa with nHA contents of 2%, 5%, and 10%, respectively. It is noteworthy that the addition of 2% nHA increased the compressive strength by more than 400%. Less dramatically, the compressive strength of the 25% hydrogel increased from 15.43 MPa to 22.21 MPa, 22.2 MPa, and 22.25 MPa with successive increases in nHA content, representing an increase of ~45% in all cases. These results can be attributed to the fact that load is transferred from the polymer matrix to the uniformly distributed nHA which prevents microcrack formation [38].In addition, hydroxyapatite reinforcing particles resist the deformation of the hydrogel. As a result, the hydrogel material demonstrated substantial anti-deformability and a marked increase in compressive strength [48]. 

It is also notable that, in the 25% scaffolds, a compressive strength of 22.2 MPa was recorded for both 5% and 10% nHA content. The increase to 10% nHA did not further improve the compressive strength of the scaffold.

### 3.3. Morphological Analyses of Nano-Hydroxyapatite (nHA) and GA/Chitosan/nHA Nanocomposite Hydrogels

**Figure 5:**
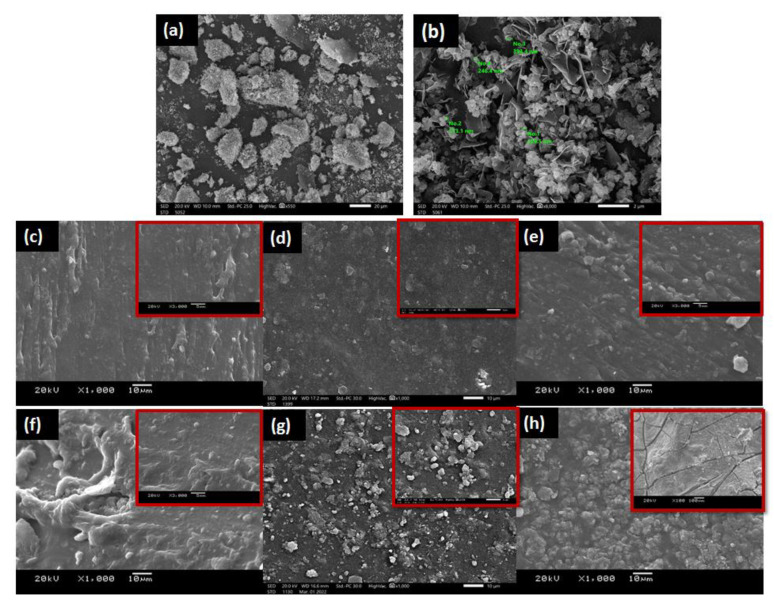
SEM images of nanohydroxyapatite (nHA) powder (**a**,**b**) with different magnifications (550×, 8000×, respectively). Surface investigations of the prepared nanocomposite hydrogels loaded with different contents of nHA: (**c**) 15% GA/chitosan/0% nHA, (**d**) 15% GA/chitosan/5% nHA, (**e**) 15% GA/chitosan/10% nHA (**f**) 25% GA/chitosan/0% nHA (**g**) 25% GA/chitosan/5% nHA (**h**) 25% GA/chitosan/10% nHa.

The morphological analyses of the nHA extracted from the bovine bone show that the particles are mostly irregular in shape. With some zooming in, the presence of rods, flakes, needles, and plate-like shapes is revealed, as shown in the SEM images in Figure 5a,b. Furthermore, nanosized particles surpass micron-sized particles in terms of high surface activity and ultrafine structures, increased bioactivity, and improved resorbability. Additional milling was used to reduce the size of the nHA particles to a nanometric size which is similar to that of human HA [49].

The high cross-linking nature of the hydrogels is confirmed by the SEM images in Figure 5. Also revealed is the compactness of the surface of the hydrogels in the gel network that increases with the increase in the GA/chitosan content. So, a more compact surface is observed in Figure 5f (25% GA/chitosan without nHA) compared to Figure 5c (15% GA/chitosan without nHA) which confirms the excellent interfacial interactions between polymers and Fe^3+^. In addition, in Figure 5d,e,g. the uniform dispersion of nHA is visible. Hydrogels containing nHA particles may have higher mechanical qualities than pure hydrogels, signifying a more favorable milieu for cell adhesion and development [9]. However, increasing the nHA to 10% is unadvisable as it leads to crack formation on the surface of the hydrogel, as can be seen in Figure 5h. This can be explained by the poor dispersion of the high nHA content relative to the GA/chitosan that resulted in a highly viscous solution and consequent agglomeration with in-site cracks, as well as the difficulty in mixing the viscous 10% composite hydrogel that caused difficulties in the application process.

### 3.4. FTIR Analysis

**Figure 6:**
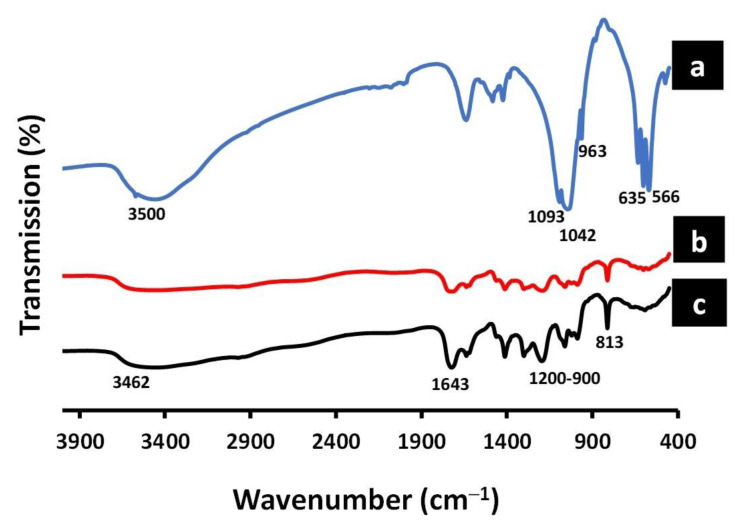
The FTIR spectrums of (**a**) nanohydroxyapatite (nHA), (**b**) 25% GA/chitosan/5% nHA hydrogel, and (**c**) 25% GA/chitosan /0% nHA hydrogel.

Figure 6a shows vibration bands characteristic of hydroxyapatite [45]. These bands appear at 3450 and 633 cm^−1^, which can be attributed to the stretching and vibration modes, respectively, of hydrogen-bond OH groups. Likewise, the bands around 1087, 961, 601, and 568 cm^−1^ can be attributed to the (PO4)^3−^ group, while the bands around 1647, 1484, and 1418 cm^−1^ can be attributed to CO_3_ ^2−^.

The FTIR spectrum for the 25% GA/chitosan hydrogels with different nHA contents (0 and 5%) are shown in Figure 6b,c, respectively. The chitosan has characteristic absorption bands at 3462–3000 cm^−1^ that can be attributed to the primary amine (NH_2_) and the (OH) group associated with the pyranose hydroxyl group. The spectrum at 1643 cm^−1^ can be attributed to the amide band (C=O) in NHCOCH_3_ groups. The bands at about 860 cm^−1^ can be attributed to the CH_3_COH group in pyranose ring vibration, which corresponds to the saccharide structure. In addition, GA has characteristic absorption bands at 3413 cm^−1^ for O-H stretching, characteristic of the glucosidic ring, and 1200–900 cm^−1^ which is the fingerprint of carbohydrates [22].

### 3.5. X-ray Diffraction (XRD) Analysis

**Figure 7:**
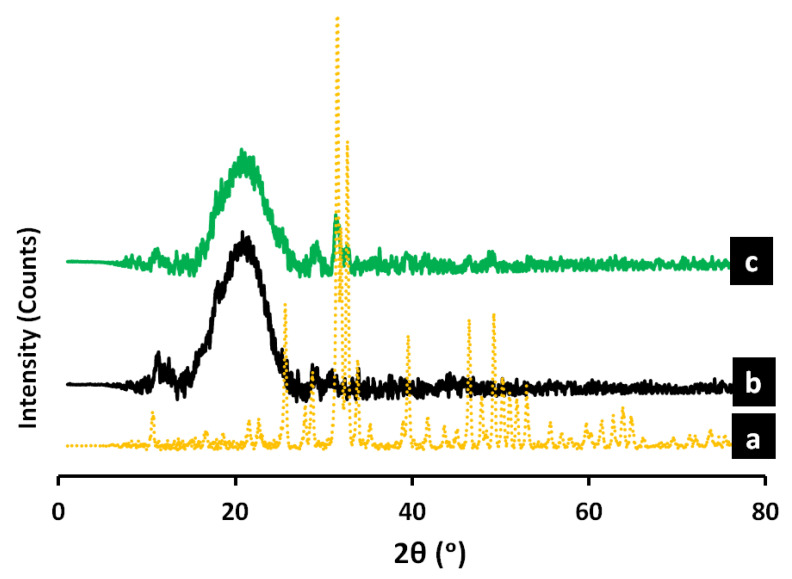
The XRD diffraction pattern of (**a**) Pure nanohydroxyapatite (nHA), (**b**) 25% GA/chitosan/0% nHA, and (**c**) 25% GA/chitosan/5% nHA composite hydrogels.

The XRD patterns of the nanohydroxyapatite (nHA) (Figure 7a) reveal peaks at 2θ = 26°, 32°, 39°, 46°, 49°, and 55°, which are consistent with the crystallinity of the HA results (JCPDS-PDF no. 74-0566). The Gum Arabic/chitosan without nHA (25% GA/chitosan/0% nHA) (Figure 7b) shows a broad peak in the range of 15°–25° (the characteristic broad peaks of GA and chitosan are around 20° and 24°, respectively), which is in full agreement with the assumption that chitosan and GA are partially crystalline polysaccharides which contain some crystalline forms embedded in the amorphous region. Figure 7c shows the addition of 5% nHA to the GA/chitosan caused peaks of nHA to appear at 26°, 32°, 39°, 46°, and 49°, which confirms the embedding of the nHA in the new GA/chitosan/5% nHA composite hydrogel. On the other hand, the characteristic peaks of GA/chitosan became broader and less intense, which may suggest the formation of a new interaction with the embedded nHA crystalline forms [20].

### 3.6. Water Absorption ASTM D570

**Figure 8:**
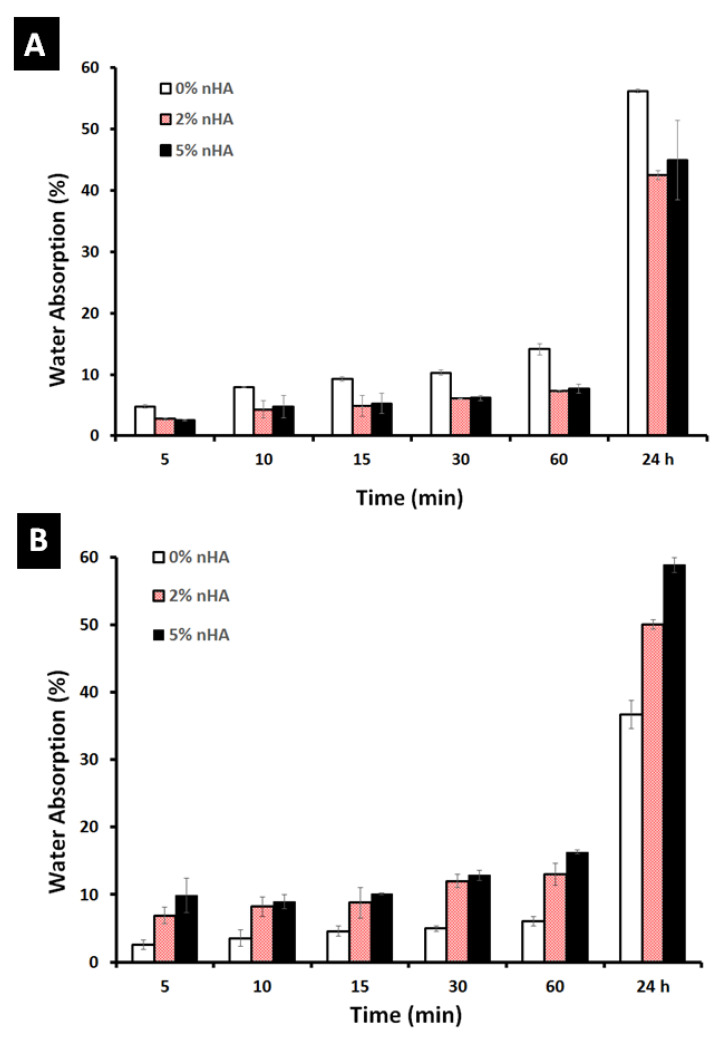
Water absorption versus time for (**A**) 15% GA/chitosan hydrogels, and (**B**) 5% GA/chitosan hydrogels with 0, 2, and 5% nHA using deionized water at room temperature.

The water absorption behavior of the hydrogels is significant during in vitro cell culture studies. The swelling of the hydrogels allows for the absorption of body fluids as well as the transfer of cell nutrients and metabolites within them. Swelling also increases the internal surface area accessible for cell infusion and adhesion by expanding the pore size of the hydrogels. Figure 8A,B depict the swelling of 15% and 25% GA/chitosan nanocomposite hydrogels with different nHA wt.% concentrations (0, 2, and 5%) in deionized water at room temperature. Due to their highly interconnected and porous nature, all the hydrogels were able to swell by absorbing water but with different percentages as a function of the time. 

As shown in Figure 8A, the swelling ratio of the 15% GA/chitosan virgin hydrogels was substantially higher than that of the hydrogels containing 2% and 5% nHA; this can be attributed to the low hydroxyl groups with lower natural polymer content that were affected by the nHA blocking [50]. After a sufficient time period of 24 h, the swelling of the hydrogels significantly increased. 

In addition, as shown in Figure 8B, the swelling ratios of the 25% GA/chitosan hydrogels were lower than the 15% GA/chitosan hydrogels (i.e., without nHA content). This can be attributed to the increase in the polymer concentration which consequently increased the cross-linking that showed lower swelling capacity than the hydrogels with low natural polymer concentration [51]. The addition of nHA positively affects the water uptake of the 25% GA/chitosan hydrogels, which may be due to the hydrophilic nature of the nanosized nHA particles as well as creating micro-voids in the high cross-linked compact GA/chitosan hydrogels. The 25% GA/chitosan hydrogels containing 5% nHA showed a swelling capacity of 60% which is higher than that of the 2% nHA which showed a swelling capacity of 50%. The hydrophilicity of the hydrogels enables the absorption of body fluid, which is mostly water, and which is necessary for nutrient and metabolite diffusion.

### 3.7. In Vitro Degradation Behavior

**Figure 9:**
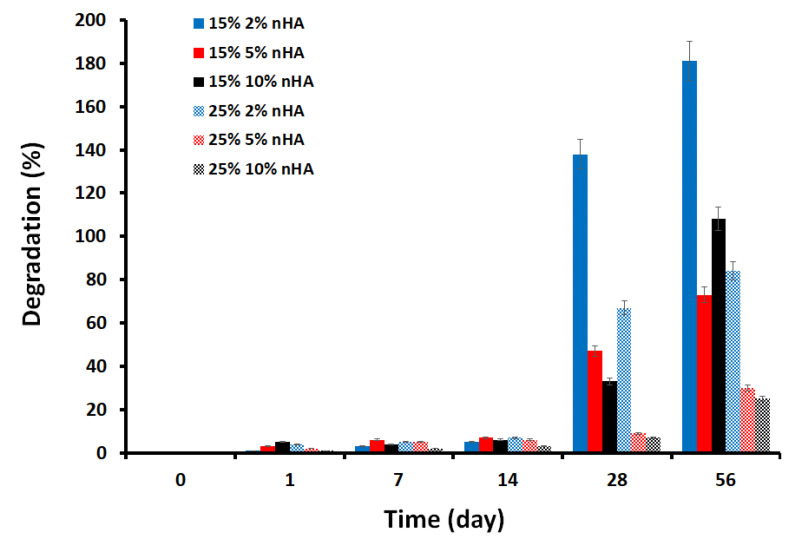
In vitro degradation of the GA/chitosan/nHA nanocomposite hydrogels in phosphate buffered saline (PBS) at 37 °C.

The physiological stability of composite hydrogels is crucial during bone regeneration; ideally, a scaffold should be degradable by endogenous enzymes or hydrolysis, synchronizing with new bone ingrowth to make enough space for new bone formation [47]. The degradation of the control and nanocomposite hydrogels in PBS solution was studied for two months. As shown in Figure 9, the highest degradation rate of 181% was observed in the 15% GA/chitosan hydrogels containing 2% nHA, while the lowest degradation rate of 30% was observed in the 25% GA/chitosan hydrogels containing 5% and 10% nHA. This may be due to the increased cross-linking of the 25% chitosan/GA/nHA nanocomposite hydrogels forming a more stable and compact hydrogel (scaffold) structure. The hydrogel’s unhurried biodegradation rate is a potentially valuable property because it helps the hydrogel to maintain its mechanical integrity while allowing enough time for bone growth to occur in the implant.

### 3.8. Apatite-Forming Ability

**Figure 10:**
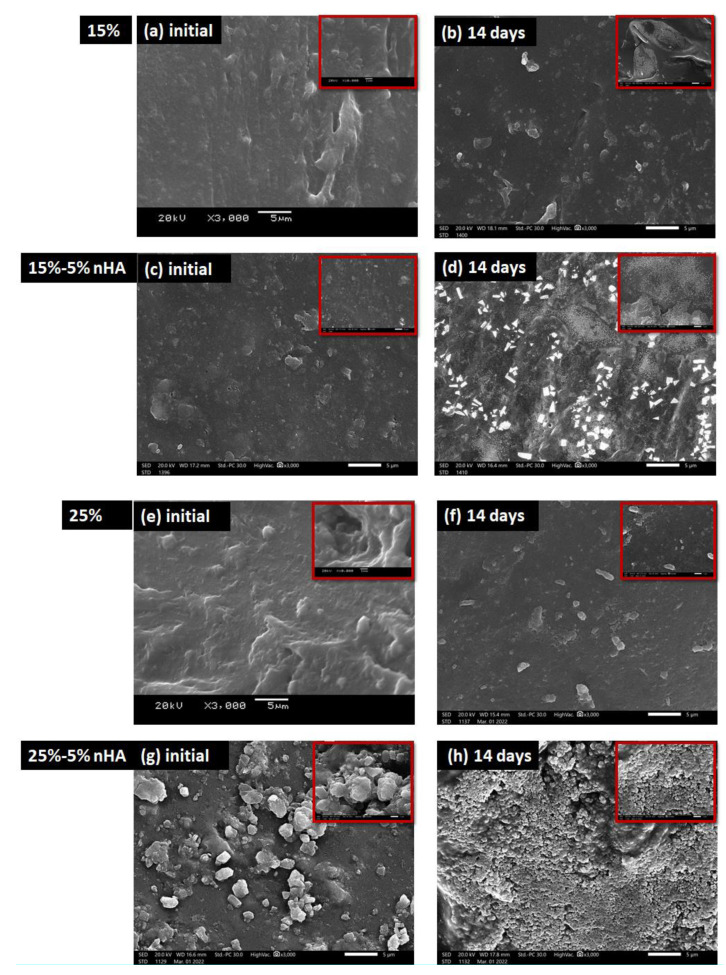
SEM images of the prepared nanocomposite hydrogels after 14 days of incubation in simulated body fluid (SBF): (**a**) 15% GA/chitosan/0% nHA initial hydrogel, (**b**) 15% GA/chitosan/0% nHA hydrogel after 14 days’ incubation time in SBF, (**c**) 15% GA/chitosan/5% nHA initial hydrogel, and (**d**) 15% GA/chitosan/5% nHA hydrogel after 14 days’ incubation time in SBF (**e**) 25% GA/chitosan/0% nHA initial hydrogel, (**f**) 25% GA/chitosan/0% nHA hydrogel after 14 days’ incubation time in SBF, (**g**) 25% GA/chitosan/5% nHA initial hydrogel, (**h**) 25% GA/chitosan/5% nHA hydrogels after 14 days’ incubation time in SBF.

After 14 days of contact of the nanocomposites with the simulated body fluid (SBF), a layer of spherical apatite particles, with diameters of approximately 1 µm to nanosized, formed on the surface of all samples of the hydrogels, as shown in Figure 10. Extensive grain agglomeration was observed on the surface layer. These particles are found on the surface of granules that have already been created. Also observed were agglomerates of new phase particles that are loosely packed, with sinuous pores of various depths and with diameters distinguishable between them. This agrees with the findings of Bai et al. [40] Apatite formation on the surface of the hydrogel has been shown to promote osteoblast proliferation and differentiation in previous studies. The ability to shape apatite can also be used to assess osteogenic bioactivity in vitro [52]. 

### 3.9. In Vivo Biocompatibility

**Figure 11:**
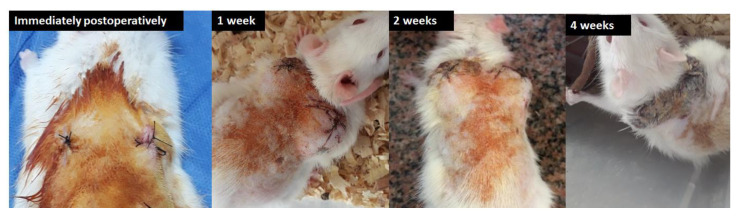
Gross clinical observations immediately postoperatively, and after intervals of one, two and four weeks.

The initial stage in evaluating the in vivo biocompatibility of an implantable material is to implant it subcutaneously or intramuscularly. Many researchers have utilized this technique to assess the biocompatibility of a biodegradable construct for tissue engineering applications [42]. In our present study, gross clinical observations did not show any mortality or signs of inflammation at the implantation site (Figure 11).

**Figure 12:**
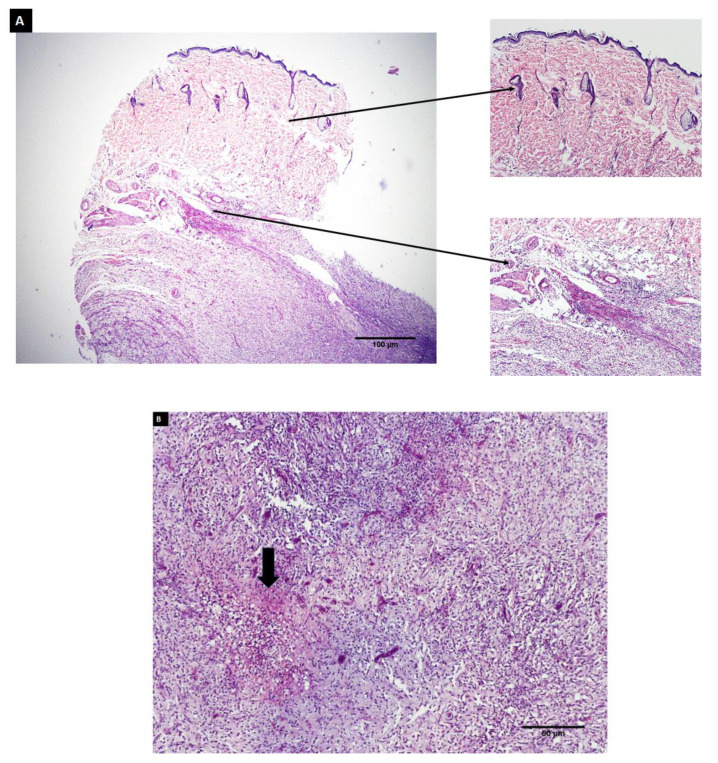
Histological sections stained with H and E of subcutaneous rat tissue surrounding implanted hydrogels (scaffolds).

Histopathological assessment was carried out to evaluate the local tissue response to subcutaneous hydrogel implantation after 4 weeks (Figure 12). The skin section stained with hematoxylin and eosin (H and E) displayed normal skin histology (Figure 12A). It revealed a cornified layer on top; below this, stratified squamous epithelial cells were observed. Skin sections also displayed hair follicles, sebaceous glands, and bundles of loose collagen fibers. The implantation of hydrogels (scaffolds) did not produce any adverse reactions. In addition, small remnants of scaffold material were seen (Figure 12B, arrow) confirming the biodegradation of the scaffold.

## 4. General Discussion

In this work, physical cross-linking was used to create nanocomposite hydrogels made up of unmodified gum arabic (GA)/chitosan with different content ratios (15, 25 and 35 wt.% of the used acrylic acid solvent), and natural nanohydroxyapatite (nHA) with different content ratios (2, 5, and 10 wt.%) for preparing novel GA/Ch/nHA nanocomposite hydrogels that can be used in bone regeneration.

The increase in GA/chitosan content ratios from 15 wt.% to 25 wt.% of the used acrylic acid solvent (i.e., without nHA content), had the advantage of significantly increasing the compressive strength from ~4 MPa to ~16 MPa (300% increase). However, the in vitro degradation decreased significantly by ~75%. Nevertheless, the scaffold’s unhurried biodegradation rate can be valuable as it helps the scaffold to maintain its mechanical integrity while allowing enough time for bone growth to occur in the implant. The reduced cross-linking of the 15% GA/chitosan hydrogels can make it suitable for injection, which may be beneficial in some clinical applications. 

The 35 wt.% GA/chitosan hydrogel is not advisable to use as it is very difficult to prepare due to the high cross-linking and compactness of the hydrogel. In addition, the compressive strength of this hydrogel is ~3 MPa which is five times less than the 25 wt.% GA/chitosan hydrogel.

Nanohydroxyapatite (nHA), [Ca_10_(PO_4_)_6_(OH)_2_], a bioactive material that is the most thermodynamically stable calcium phosphate salt, can increase the concentration of local Ca^2+^ that activates the proliferation of osteoblasts and also simultaneously increase the mechanical properties of the hydrogel biomaterial. However, an excessive nHA concentration of 10% in the 25% GA/chitosan hydrogel did not significantly affect the compressive strength and adversely caused crack formation and agglomeration as seen in the SEM images, while also causing difficulty in the preparation of the hydrogel due to excessive viscosity. This is also in line with previous studies which found that cells seem to prefer the tender component of the scaffold which could be responsible for the first cell anchorage, rather than the stiffer component [49].

Future developments of this study will aim at direct 3D printing of the nanocomposite hydrogels which will be able to be designed for specific defects in the patient’s bone and exactly match the contours of surrounding tissues. The printed scaffold would offer the combined advantages of high strength, bioactivity, ease of surgical handling, improved load support, and better functionality after implantation [28].

## 5. Conclusions

In this work, physically cross-linked nanocomposite hydrogels composed of unmodified gum arabic (GA), chitosan (Ch), and natural nanohydroxyapatite (nHA) with different content ratios were successfully synthesized. The unmodified GA was successfully used without any further changes which opens up new possibilities for the use of GA-based hydrogels in bone regeneration. The natural nHA was successfully dispersed and positively impacted the mechanical properties of the hydrogel in which the synthesized GA/Ch/nHA nanocomposite hydrogels exhibited compressive strength of 15.43–22.20 MPa which is similar to that of the natural cortical bone.

The in-vitro and in-vivo analyses confirmed the biodegradation of the novel GA/Ch/nHA nanocomposite hydrogels and the lack of allergic reaction to them when implanted subcutaneously. According to these findings, the GA/Ch/nHA nanocomposite hydrogels are recommended for preparing bioactive nanoscaffolds for testing in bone regeneration applications.

## Figures and Tables

**Table 1:** The order of chemicals for the simulated body fluid (SBF) preparation.

Order	Reagent	Amount (g)
1	NaCl	7.996
2	NaHCO_3_	0.350
3	KCl	0.224
4	K_2_HPO_4_·3H_2_O	0.228
5	MgCl_2_·6H_2_O	0.305
6	1 kmol/m^3^ HCl	40 cm^3^
7	CaCl_2_	0.278
8	Na_2_SO_4_	0.071
9	(CH_2_OH)_3_CNH_2_	6.057
10	1 kmol/m^3^ HCl	Appropriate amount for adjusting pH

## Data Availability

Data are contained within the article or supplementary material.

## Supplementary Materials

The following supporting information can be downloaded at: https://www.mdpi.com/article/10.3390/polym14153052/s1, Figure S1: Preparation steps for nanohydroxyapatite (nHA); Figure S2: Stress- strain compressive strength curves of the prepared hydrogels.
