# Supplementary material for: Unmodified Gum Arabic/Chitosan/Nanohydroxyapatite Nanocomposite Hydrogels as Potential Scaffolds for Bone Regeneration
Source: Polymers (Basel). 2022 Jul 28;14(15):3052. doi: 10.3390/polym14153052 (PMC9370697; doi:10.3390/polym14153052)
Supplement: Supplementary file 1 [file polymers-14-03052-s001.zip › polymers-1803191-supplementary.pdf]

<u>Supplementary Material</u>

Unmodified Gum Arabic/Chitosan/Nanohydroxyapatite Nanocomposite Hydrogels as Potential Scaffolds for Bone Regeneration

Lara E. Makar [1,*], Norhan Nady [2,*], Ahmed Abd El-Fattah [1,3], Neivin Shawky [4] and Sherif H. Kandil [1]

[1] Department of Materials Science, Institute of Graduate Studies and Research, Alexandria University,
El-Shatby, Alexandria 21526, Egypt; a_abdelfattah@alexu.edu.eg (A.A.E.-F.); s.kandil@usa.net (S.H.K.)
[2] Polymeric Materials Research Department, City of Scientific Research and Technological Applications (SRTA-City), Alexandria 21934, Egypt
[3] Department of Chemistry, College of Science, University of Bahrain, Sakhir P.O. Box 32038, Bahrain
[4] Oral and Maxillofacial Surgery Department, Faculty of Dentistry, Alexandria University, Champollion Street—Azarita, Alexandria 21526, Egypt; noovy60@hotmail.com
* Correspondence: laramakar.dent@gmail.com or igsr.lara@alexu.edu.eg (L.E.M.); norhan.nady77@yahoo.com (N.N.); Tel.: +20-1227289592 (L.E.M.); +20-1090918521 (N.N.)

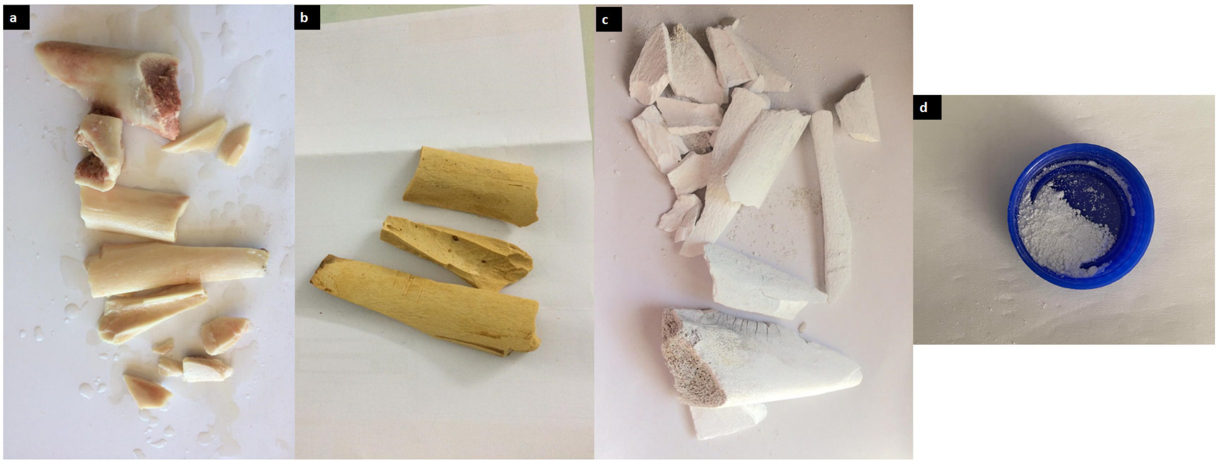

Figure S1. Preparation steps for nanohydroxyapatite (nHA). (a). Th**e** bovine bone was washed carefully with water and acetone to remove the fats and other impurities. (b). The bone was dried at 160 °C for 48 h. (c). The bones were annealed in an electric furnace at 750°C for 6. (d). the bone was ground with mortar and pestle to a particle size less than 450 μm

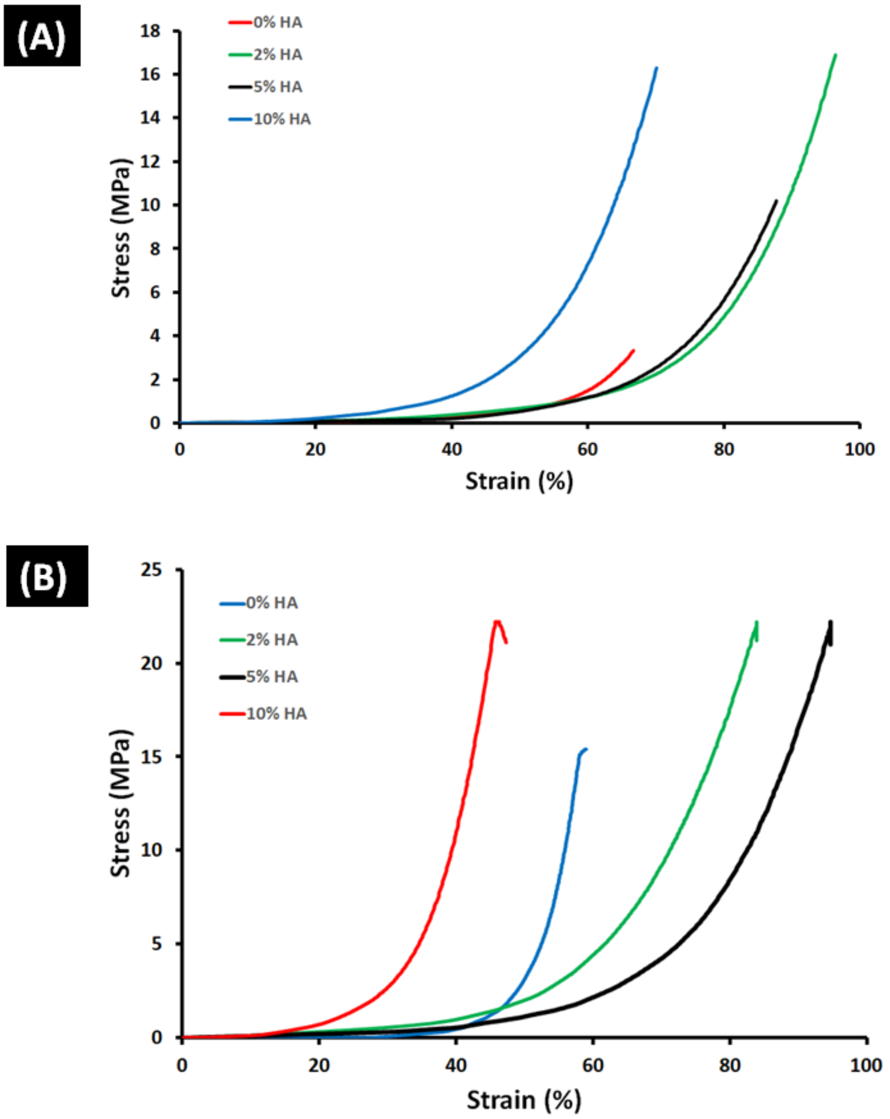

Figure S2. Stress–strain compressive strength curves of prepared hydrogels: (**A**) GA/chitosan (15%), and (**B**) GA/chitosan (25%), with different nHA contents (0, 2, 5, and 10%, respectively).
